# Circulation of Extended-Spectrum Beta-Lactamase-Producing *Escherichia coli* of Pandemic Sequence Types 131, 648, and 410 Among Hospitalized Patients, Caregivers, and the Community in Rwanda

**DOI:** 10.3389/fmicb.2021.662575

**Published:** 2021-05-14

**Authors:** Elias Eger, Stefan E. Heiden, Katja Korolew, Claude Bayingana, Jules M. Ndoli, Augustin Sendegeya, Jean Bosco Gahutu, Mathis S. E. Kurz, Frank P. Mockenhaupt, Julia Müller, Stefan Simm, Katharina Schaufler

**Affiliations:** ^1^Pharmaceutical Microbiology, Institute of Pharmacy, University of Greifswald, Greifswald, Germany; ^2^Institute of Bioinformatics, University Medicine Greifswald, Greifswald, Germany; ^3^College of Medicine and Health Sciences, University of Rwanda, Kigali, Rwanda; ^4^University Teaching Hospital of Butare, Butare, Rwanda; ^5^Institute of Tropical Medicine and International Health, Charité Medical University of Berlin, Berlin, Germany

**Keywords:** ESBL—*E. coli*, whole-genome sequencing, Rwanda, virulence factors, phylogenetic analysis

## Abstract

Multi-drug resistant (MDR), gram-negative *Enterobacteriaceae*, such as *Escherichia coli* (*E. coli*) limit therapeutic options and increase morbidity, mortality, and treatment costs worldwide. They pose a serious burden on healthcare systems, especially in developing countries like Rwanda. Several studies have shown the effects caused by the global spread of extended-spectrum beta-lactamase (ESBL)-producing *E. coli*. However, limited data is available on transmission dynamics of these pathogens and the mobile elements they carry in the context of clinical and community locations in Sub-Saharan Africa. Here, we examined 120 ESBL-producing *E. coli* strains from patients hospitalized in the University Teaching Hospital of Butare (Rwanda), their attending caregivers as well as associated community members and livestock. Based on whole-genome analysis, the genetic diversification and phylogenetics were assessed. Moreover, the content of carried plasmids was characterized and investigated for putative transmission among strains, and for their potential role as drivers for the spread of antibiotic resistance. We show that among the 30 different sequence types (ST) detected were the pandemic clonal lineages ST131, ST648 and ST410, which combine high-level antimicrobial resistance with virulence. In addition to the frequently found resistance genes *bla*_*CTX–M–15*_, *tet*(34), and *aph(6)-Id*, we identified *csg* genes, which are required for curli fiber synthesis and thus biofilm formation. Numerous strains harbored multiple virulence-associated genes (VAGs) including *pap* (P fimbriae adhesion cluster), *fim* (type I fimbriae) and *chu* (Chu heme uptake system). Furthermore, we found phylogenetic relationships among strains from patients and their caregivers or related community members and animals, which indicates transmission of pathogens. Also, we demonstrated the presence and potential transfer of identical/similar ESBL-plasmids in different strains from the Rwandan setting and when compared to an external plasmid. This study highlights the circulation of clinically relevant, pathogenic ESBL-producing *E. coli* among patients, caregivers and the community in Rwanda. Combining antimicrobial resistance with virulence in addition to the putative exchange of mobile genetic elements among bacterial pathogens poses a significant risk around the world.

## Introduction

The versatility of *Escherichia coli* (*E. coli*) is based on the diversity of genetic substructures within this species (Whittam et al., 1983). In addition to commensal strains, which are an essential part of the non-anaerobic intestinal microflora of humans, other mammals and birds, pathogenic variants occur. The dissimilarity of these pathotypes depends also on their virulence attributes, resulting in a wide range of pathologies in both humans and animals. The intestinal pathogenic *E. coli* (InPEC) express characteristic virulence factors that allow to adhere and invade intestinal cells, causing specific enteric and diarrheal diseases. While InPEC are obligate pathogens, extraintestinal pathogenic *E. coli* (ExPEC) are part of the intestinal microbiome but exhibit a heterogeneous composition of virulence factors to colonize niches such as the urinary tract (Kaper et al., 2004). They can thus cause infections in almost any organ or non-intestinal site, regardless of the state of the host’s immune system (Russo and Johnson, 2000). However, a strict differentiation of pathogenic and commensal *E. coli* is difficult, provided by their rapid geno- and phenotypic adaptation to changing environmental conditions, for example through horizontal gene transfer (Pallen and Wren, 2007). Despite the plasticity of the genome, phylogenetic studies have shown some clonality within the population structure of *E. coli*, from which seven distinct phylogenetic groups were derived (Jaureguy et al., 2008; Touchon et al., 2009; Clermont et al., 2013). Usually, commensal strains and obligatory pathogens belong to the phylogroups A and B1, whereas strains with extended virulent attributes (mainly ExPEC) are part of the phylogroups B2, D, and F, with the latter as a sister group of B2 (Escobar-Páramo et al., 2004; Clermont et al., 2013). Multi-locus sequence typing (MLST) allows additional classification and several phylogenetic studies suggest the spread of pandemic high-risk clonal lineages including primarily sequence type (ST) 131 (Nicolas-Chanoine et al., 2008; Ewers et al., 2010; Hussain et al., 2012), ST648 (Ewers et al., 2014; Schaufler et al., 2019), ST410 (Schaufler et al., 2016b; Zurita et al., 2020), putatively ST405 (Manges et al., 2019), and others.

The management of zoonotic infections caused by antibiotic-resistant bacteria has become a multidisciplinary challenge for all modern healthcare systems and is nowadays often approached in a holistic One Health context. Bacterial pathogens spread through direct contact among humans and animals, indirectly by (environmental) pollution and also through non-living and living vectors (Rahman et al., 2020). One example for the latter are houseflies, which have been demonstrated to carry antibiotic-resistant pathogens including extended-spectrum beta-lactamases-(ESBL)-producing *E. coli* (Rahuma et al., 2005; Heiden et al., 2020b; Tufa et al., 2020) non-susceptible to third-generation cephalosporins (e.g., cefotaxime) and monobactams (e.g., aztreonam) (Bevan et al., 2017). Notably, ESBL enzyme production is often accompanied by cross- and co-resistances (Cantón and Coque, 2006; Hidron et al., 2008; Pitout, 2012) resulting in multi-drug resistant (MDR) representatives (Beceiro et al., 2013).

Both pandemic clonal lineages including the aforementioned ST131, ST648 and others, as well as mobile genetic elements (i.e., ESBL-plasmids) drive the spread of antibiotic resistance and virulence-associated genes (VAGs) (Cantón and Coque, 2006). Interestingly, previous studies suggest that ESBL-plasmid carriage does not ineluctably reduce bacterial fitness, which seems particularly true for specific clonal lineages (McNally et al., 2016; Schaufler et al., 2016a; Ranjan et al., 2018; Monárrez et al., 2019; Schaufler et al., 2019). The combination of MDR with high-level bacterial virulence and fitness leads to the emergence of these pandemic, high-risk clonal lineages and subsequently contributes to treatment failures, increasing morbidity, and mortality (Melzer and Petersen, 2007; Schwaber and Carmeli, 2007; Tumbarello et al., 2007; Beceiro et al., 2013; Schaufler et al., 2019; Heiden et al., 2020a).

The One Health concept—addressing human, animal and environmental health—encounters some challenges, especially in low-income countries like Sub-Saharan Africa/Rwanda. On the one hand, the lack of surveillance systems may result in inadequate establishment and implementation of hygienic strategies and therapy guidelines (Muvunyi et al., 2011; Ntirenganya et al., 2015; Carroll et al., 2016). On the other hand, uncontrolled over-the-counter sale of partially counterfeit and substandard antibiotic drugs (Kayumba et al., 2004; Carroll et al., 2016) as well as close human-livestock contact and household crowding might contribute to the broad occurrence and interspecies transmission of MDR bacteria in Sub-Saharan Africa.

This study aimed to investigate whether (i) ESBL-producing *E. coli* circulate among patients, caregivers, the community, and animals in Rwanda, (ii) some of these belong to pandemic high-risk clonal lineages and how they are phylogenetically related, (iii) they demonstrate virulence-associated features, (iv) their mobile genetic elements contribute to the spread of antibiotic resistance.

## Materials and Methods

### Bacterial Strains

The *E. coli* strains investigated in this study were sampled over a time period of 8 weeks at the University Teaching Hospital of Butare (Rwanda) in 2014 (previously described by Kurz et al., 2017). Rectal swabs (Sarstedt AG & Co. KG, Nümbrecht, Germany) were collected from patients and caregivers at admission and discharge as well as from several community members and animals. Each patient had their own caregiver, who were usually relatives accompanying the patient upon admission. They stayed in the patient’s room and were involved in personal care of the patient and food preparation. This is a common practice in African hospitals (Hoffman et al., 2012; Ugochukwu, 2013). Sample groups consisting of a patient and related caregiver, and associated family members, neighbors and/or pets were included in the same study-ID. The samples were plated onto chromogenic agar (CHROMagar-ESBL, Mast Diagnostica GmbH, Reinfeld, Germany) supplemented with 2 μg/mL cefotaxime (Cayman Chemical Company, Ann Arbor, United States) and incubated at 37°C. For putative ESBL-positive colonies, the production of ESBL and/or ampicillinase (AmpC) was verified (ESBL-AmpC-Detection Test, Mast Diagnostica GmbH, Reinfeld, Germany) and all strains positive for AmpC only were excluded. The strains were stored at –80°C in LB broth (Carl Roth GmbH & Co. KG, Karlsruhe, Germany) supplemented with 20% (V/V) glycerol (Merck KGaA, Darmstadt, Germany). Originally, we have obtained overall 289 ESBL-producing *E. coli* strains (from patients, caregivers, community members, and animals), with 120 selected strains (based on related study-IDs) that were whole-genome sequenced. Additionally, flies caught with fly traps at different wards of the hospital examined in a previous study (Heiden et al., 2020b) were partly included in this study (Supplementary Table 1).

### Whole-Genome Sequencing

One single *E. coli* colony was cultured in LB broth supplemented with 2 μg/mL cefotaxime overnight and the total DNA was extracted using the MasterPure^TM^ DNA Purification Kit for Blood, Version II (Lucigen, Middleton, United States) according to the manufacturer’s instructions. DNA was purity-controlled and quantified using NanoDrop^TM^ 2000 (Thermo Fisher Scientific Inc., Waltham, United States). WGS was performed in collaboration with LGC (LGC Genomics GmbH, Berlin, Germany) with 150 bp paired-end-reads using Illumina NextSeq 500/550 V2.

### Genomic Analysis

Raw reads were quality-trimmed, adapter-trimmed and contaminant-filtered using BBDuk from BBTools v. 38.86^1^. After *de novo* assembly (at a maximum coverage of 100×) using shovill v. 1.1.0^2^ in combination with SPAdes v. 3.14.1 (Bankevich et al., 2012), draft genomes were polished by mapping all trimmed reads back to the contigs with bwa v. 0.7.17 (Li and Durbin, 2009), processing SAM/BAM files marking optical duplicates with Samtools v. 1.10 (Li et al., 2009) and calling variants with Pilon v. 1.23 (Walker et al., 2014) (Supplementary Table 2).

Plasmid sequences of all strains were manually extracted using similarity searches (BLASTN Megablast) against the NCBI nucleotide collection for visualization in BRIG v. 0.95-dev.0004 (Alikhan et al., 2011). The *in silico* MLST, antibiotic resistance/virulence gene and single-nucleotide polymorphism (SNP) detection was carried out using mlst v. 2.19.0^3^, ABRicate v. 1.0.0^4^, and snippy v. 4.6.0^5^. We inferred core SNP phylogenies for pandemic sequence types. Alignments were filtered for recombinations using Gubbins v. 2.4.1 (Croucher et al., 2015) and core SNPs extracted using snp-sites v. 2.5.1 (Page et al., 2016) [core SNP sites filtered out: 1,219 (ST131); 0 (ST354); 5,370 (ST405); 2,940 (ST410); 3,564 (ST648)]. The final core SNP alignments contained 208 (ST131; reference: PBIO440), 20 (ST354; reference: PBIO388), 177 (ST405; reference: PBIO397), 154 (ST410; reference: PBIO289), and 135 (ST648; reference: PBIO368) sites (Supplementary Table 3). Maximum likelihood trees were inferred with RAxML-NG v. 1.0.0 (Kozlov et al., 2019) using GTR + G (discrete GAMMA model of rate heterogeneity with 4 categories) and searching from 500 random and 500 parsimony-based starting trees. The best-scoring maximum likelihood tree supplemented with support values from 1,000 non-parametric bootstrap replicates was midpoint-rooted and visualized with iTOL v. 5.7 (Letunic and Bork, 2019). To assess the population structure of all available genomes, we inferred an additional phylogeny constructed with JolyTree v 2.0.19092ac (Criscuolo, 2019) of all 120 strains from this study and 13 housefly isolates previously published (Heiden et al., 2020b). A synteny plot comparing ST38 chromosome- and plasmid-derived contigs was created with genoPlotR v 0.8.9 (Luan and Li, 2004).

### Minimum Inhibitory Concentration of Colistin

When the genotype was positive for colistin resistance (presence of mcr genes), we evaluated the resistance phenotype by determining the minimum inhibitory concentration (MIC) using MICRONAUT MIC-Strip Colistin (Merlin Diagnostika GmbH, Bornheim, Germany) according to the manufacturer’s instructions and interpreted the results according to the published breakpoints of EUCAST (The European Committee on Antimicrobial Susceptibility Testing, 2021). Experiments were performed thrice.

## Results

### Phylogenetic Grouping and Multi-Locus Sequence Typing

**FIGURE 1:**
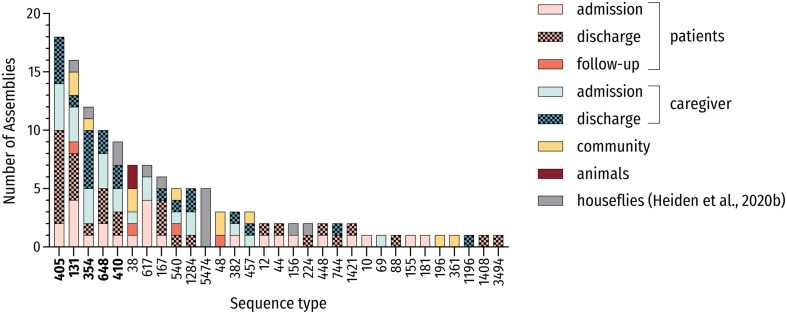
MLST-based distribution of investigated strains. Distribution of all investigated strains including genomes of ESBL-producing *E. coli* carried by houseflies (Heiden et al., 2020b; *n* = 133) and associated sequence types (STs). The five most prevalent STs are in bold.

The largest fraction of the 120 ESBL-producing *E. coli* originated from rectal swabs of patients (50.8%; 61/120), followed by caregivers (38.3%; 46/120), neighbors (5.0%; 6/120), family members (4.2%; 5/120), and animals (1.7%; 2/120) (Figure 1 and Supplementary Table 1). The majority of strains belonged to phylogroup A (30.8%; 37/120), which usually comprises commensal strains. The remaining strains were distributed among the phylogroups D (21.7%; 26/120), F (20.0%; 24/120), and B2 (14.2%; 17/120) as well as phylogroups B1 (6.7%; 8/120) and C (6.7%; 8/120), based on Clermont’s revised *E. coli* phylotyping method (Clermont et al., 2013).

Multi-locus sequence typing (MLST) from genomic data revealed 30 different STs. In total, sixty percent (18/30) of identified STs were present more than once and 10 or more strains belonged to 4 main STs (13.3%; 4/30) including the high-risk ST131 and ST648 *E. coli* lineages. These most frequently encountered STs were ST405 (15.0%; 18/120), ST131 (12.5%, 15/120), ST354 (9.2%; 11/120) and ST648 (8.3%; 10/120), which accounted for 45.0% (54/120) of all strains. Including the pandemic ST410 lineage, more than half of all strains (50.8%; 61/120) belonged to one of the five most prevalent STs in this study (Figure 1). These sequence types belonged to phylogroups B2 (ST131), D (ST405), and F (ST354, ST648) as well as phylogroup C (ST410), thus underlining the heterogeneity of phylogenetic backgrounds among ESBL-producing *E. coli*.

### Phylogenetic Relationships

**FIGURE 2:**
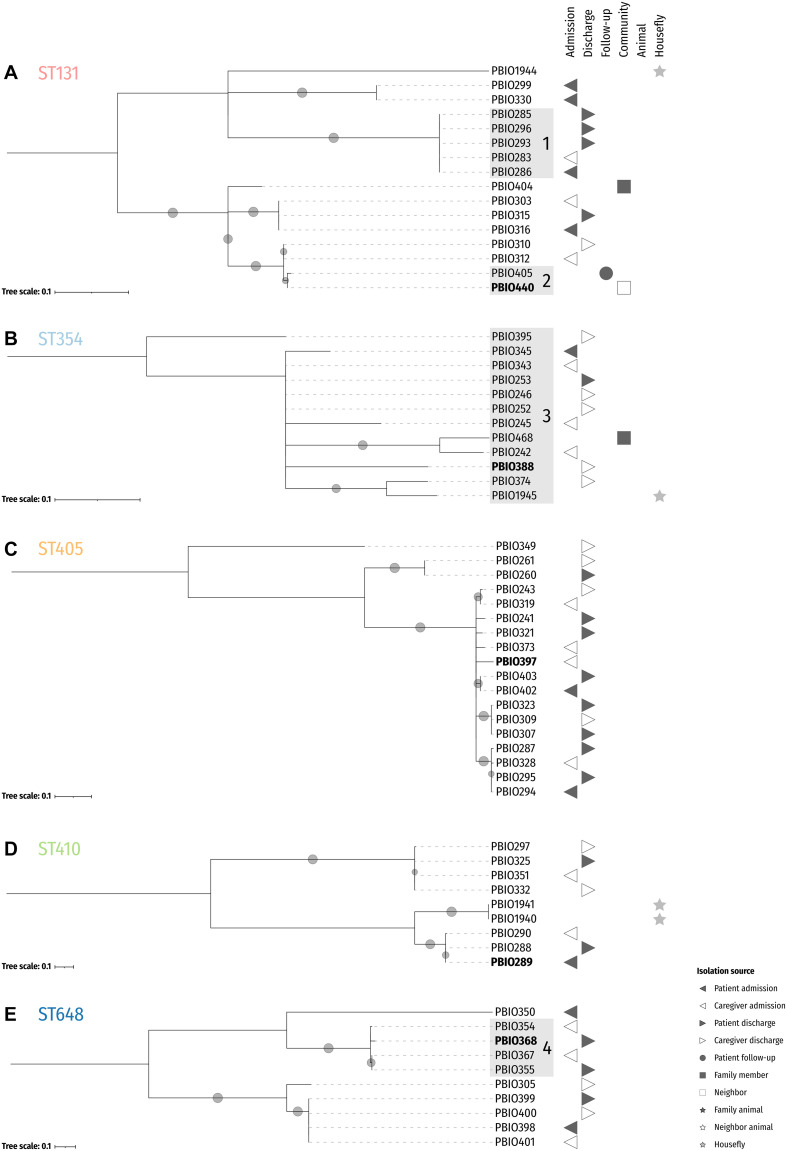
Midpoint-rooted maximum likelihood core SNP phylogenies of the five dominating sequence types. Reference isolates for ST131 (PBIO440; **A**), ST354 (PBIO388; **B**), ST405 (PBIO397; **C**), ST410 (PBIO289; **D**), and ST648 (PBIO368; **E**) are highlighted in bold. The trees are based on alignments with 208 **(A)**, 20 **(B)**, 177 **(C)**, 154 **(D)**, and 135 **(E)** sites. Sub-clades are highlighted in gray. Circles at branches display bootstrap proportions ≥50% (1,000 replicates). Symbols besides the tree depict isolation sources as given in the legend.

To elucidate phylogenetic relationships, we constructed a tree in an alignment-free manner for all investigated genomes (Supplementary Figure 1). Additionally, we inferred phylogenies, which are based on SNPs in the core genome of strains belonging to the five predominant STs of this study to assess potential transmission scenarios (Figure 2). For comparative reasons, we included 13 genomes of ESBL-producing *E. coli* isolated from houseflies (Heiden et al., 2020b) originating from the same Rwandan hospital.

The phylogenetic analysis (Supplementary Figure 1) shows that the *E. coli* strains were distributed among six distinct phylogroups and grouped into several clades according to their sequence type. Within these ST-associated phylogenies, several sub-clades were defined with genomes interspersed in patients, caregivers, related community members as well as animals and flies, which suggests common phylogenetic backgrounds (Clermont et al., 2011) potentially based on interspecies transmission. For example, PBIO458 (study-ID 60) and PBIO459 [(study-ID 133)—both ST38 isolated from two animals—clustered with four different strains from patients, family members and neighbors, PBIO272 (patient admission, study-ID 60), PBIO451 (neighbor, study-ID 60), PBIO455 (family member, study-ID 60), and PBIO467 (patient follow-up, study-ID 60)]. These findings are corroborated by results of two of our previous studies, where we demonstrated the likely transmission of ESBL-producing *E. coli* ST38 among humans and animals (Guenther et al., 2017; Schaufler et al., 2018). Note, however, that in this current study, only one representative of ST38 [PBIO302 (caregiver admission, study-ID 131)] carried a chromosomally encoded *bla*_CTX–M–15_ gene and the before mentioned strains carried plasmid-encoded ESBLs (Supplementary Figure 1), which is contrary to our previous findings. We then compared the *bla*_CTX–M–15_ gene-carrying chromosomal contig of PBIO302 to two of the plasmid-encoded *bla*_CTX–M–15_ sequences of ST38 (PBIO272 and PBIO459; Supplementary Figure 2). The chromosomal sections of PBIO302, PBIO272, and PBIO459 were highly similar, except the chromosomal insertion of *bla*_CTX–M–15_ in PBIO302. This resistance gene was flanked by transposable elements, as described below.

In addition, strains with the numbers PBIO1939, PBIO1942, PBIO1945, PBIO1946, and PBIO1947 from houseflies were in the same sub-clade as strains isolated from different human sources indicating the potential role of living vectors in the spread of pathogenic bacteria.

For the phylogenetic trees of the five predominant STs of this study (Figure 2) it is interesting to notice that some genomes stemming from different sources were more closely related than genomes from the same source. For example, PBIO283 [(study-ID 92) Figure 2A, ST131, sub-clade 1], which originates from a caregiver at admission differed in 0.2 ± 0.0003 SNPs/Mbp with strains isolated from the related patient at admission [PBIO286 (study-ID 92)] as well as discharge [PBIO285 (study-ID 92)] and unrelated patients at discharge [PBIO293 (study-ID 114) and PBIO296 (study-ID 117)]. Moreover, PBIO440 [(study-ID 133) Figure 2A, ST131, sub-clade 2], isolated from a community member, only varied in 0.2 SNPs/Mbp compared to a follow-up strain of an already discharged patient [PBIO405 (study-ID 434)]. Notably, all strains belonging to ST354 (Figure 2B, sub-clade 3) only differed in 1.0 ± 0.4 SNPs/Mbp including one strain carried by a housefly (PBIO1945). Also interesting was the difference between PBIO368 [(study-ID 335) Figure 2E, ST648, sub-clade 4], originating from a patient at discharge, and strains of distinct sources [PBIO354 (caregiver admission, study-ID 265), PBIO355 (patient discharge, study-ID 265), and PBIO367 (caregiver admission, study-ID 288)], differing in 0.9 ± 0.1 SNPs/Mbp. These numbers of SNPs were up to 10-fold lower than described for clonal EHEC strains during an outbreak in Germany (1.8 SNPs/Mbp) (Grad et al., 2012; Been et al., 2014), suggesting the circulation of only a handful of sequence types in this African setting, which interestingly happen to mostly be international high-risk clonal lineages. In addition, some strains from identical STs were carried by both flies and humans, for example PBIO1945 and PBIO374 (caregiver discharge, study-ID 401; Figure 2B), again indicating the role of flies in the spread of antibiotic-resistant pathogens.

### Antimicrobial Resistance Determinants

The predominant ESBL-gene was *bla*_CTX–M–15_ (87.5%; 105/120). Furthermore, 76 strains (63.3%; 76/120) carried *bla*_OXA–1_ and eight (6.7%; 8/120) *bla*_CTX–M–27_. Previous studies reported the co-occurrence of *bla*_CTX–M–15_ and *bla*_OXA–1_, whereas other members of the CTX-M family (e.g., *bla*_CTX–M–27_) and *bla*_OXA–1_ appear to be mutually exclusive (Schaufler et al., 2016b; Livermore et al., 2019; Bodendoerfer et al., 2020). Here, almost all strains carrying the *bla*_OXA–1_ gene (98.7%; 75/76) carried the *bla*_CTX–M–15_ gene in addition and none showed the combination of *bla*_OXA–1_ and another CTX-M gene. The co-occurence of distinct CTX-M genes was not detected. For the majority of *bla*_CTX–M_-positive strains (71.1%; 81/114), the corresponding gene was encoded on a plasmid (Supplementary Figure 1). Interestingly, all strains of phylogroup F (20.0%; 24/120) were carriers of *bla*_CTX–M–15_ genes, of which 87.5% (21/24) were located on the chromosome (all strains belonging to ST648 and ST354). These chromosomally encoded genes were flanked by an insertion sequence IS*Ecp1* upstream and a Tn*2* downstream. Also note that the strains PBIO242 and PBIO245 (ST354) showed two of these motifs consecutively. The *bla*_CTX–M–15_ gene of the ST410 strains PBIO288, PBIO289, and PBIO290 were found in proximity to an upstream-located IS*Ecp1* only. Previous studies have already demonstrated the diversity and global distribution of these complex transposable units in enterobacteria (Poirel et al., 2005; Lartigue et al., 2006; Decano and Downing, 2019; Ludden et al., 2020; Yoon et al., 2020).

Genes encoding for carbapenem-hydrolyzing enzymes, like *bla*_OXA–48_ or *bla*_NDM–1_, were not present.

A growing body of ESBL-producers is MDR (Cantón and Coque, 2006; Hidron et al., 2008; Pitout, 2012; Beceiro et al., 2013). In our study, all investigated strains (100.0%; 120/120) were carriers of genes conferring resistance to three or more different classes of antimicrobial agents, thus exhibiting a MDR genotype (Magiorakos et al., 2012). In total, 120 strains (100.0%; 120/120) carried resistance genes to tetracyclines (*tet*), followed by genes encoding for aminoglycoside (*aac, aad*, and *aph* [98.3%; 118/120]), sulfonamide [*sul* (90.0%; 108/120)], trimethoprim [*dfr* (89.2%; 107/120)], chloramphenicol [*cat* (72.5%; 87/120)], and fluoroquinolone [*aac(6′)-Ib-cr* and *qnr* (71.7%; 86/120)] resistances. Two strains (1.7%; 2/120) belonging to ST181 and ST540 were carriers of *mcr-9* but showed phenotypic susceptibility to colistin, which is a last-resort antibiotic (MIC of both strains: 0.5 μg/mL). Interestingly, previous studies suggest that this latest member of the *mcr* gene family does not always confer phenotypic resistance to colistin in clinical isolates, although overexpression in *E. coli* TOP10 cells leads to increased MICs (Carroll et al., 2019; Kieffer et al., 2019; Tyson et al., 2020).

In addition to the resistances described, genes encoding for efflux pumps were found frequently, with *mdfA* in all (120/120) and *acrB* in 85.8% (103/120) of all genomes.

### Virulence-Associated Genes

**FIGURE 3:**
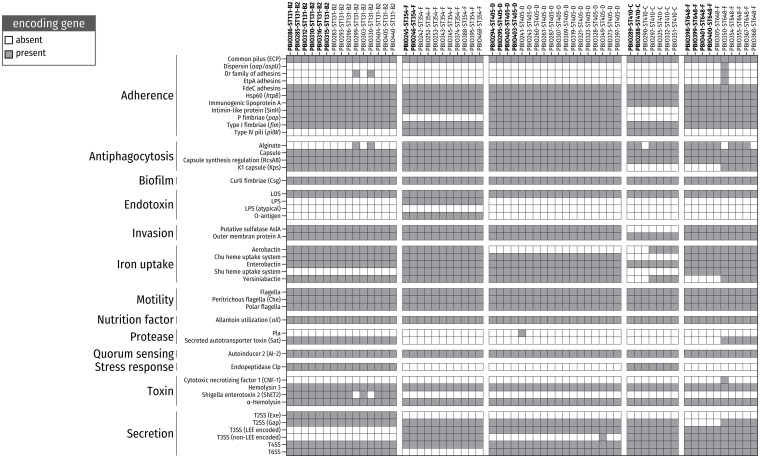
Presence/absence of virulence factors (VAGs) among ESBL-producing *E. coli* belonging to the five most prevalent sequence types (STs). Color-filled boxes show the presence of genes (coverage and identity ≥ 65%) encoding for different VAGs.

As previously demonstrated by us and others, the combination of MDR and high-level bacterial virulence seems to be a hallmark of pandemic high-risk clonal lineages (Hussain et al., 2012; Ewers et al., 2014; Shaik et al., 2017; Schaufler et al., 2019). To investigate the strains’ genetic virulome, we analyzed the genomes of the predominant ST131 (*n* = 15), ST648 (*n* = 10), ST354 (*n* = 11), ST405 (*n* = 18), and ST410 (*n* = 7) for these features (Figure 3).

The strains belonging to the five predominant STs carried several VAGs, mainly associated with adherence, antiphagocytosis, biofilm formation, invasion, iron uptake and bacterial secretion. The ability to attach to surfaces/cells and form biofilms is a common strategy used by bacterial populations to resist antibiotic treatment and host defense mechanisms as well as cause infection (Moser et al., 2017; Amanatidou et al., 2019). In particular, genes for the P fimbriae adhesion cluster [*pap* operon (70.5%; 43/61)], Dr family of adhesins (4.9%; 3/61) and type I fimbriae [*fim* (100%; 61/61)], which are necessary for uroepithelia cell adhesion and invasion, and, thus, for causing urinary tract infection (Mulvey, 2002), were frequently found. Notably, strains belonging to ST354 and ST410 showed a lack of *pap* genes, which is consistent with previous findings (Vangchhia et al., 2016; Zogg et al., 2018; Schaufler et al., 2019). Furthermore, we detected several members of the *csg* gene family in all genomes (100%; 120/120). These genes encode curli fibers, which are essential components of bacterial biofilms (Hammar et al., 1995; Evans and Chapman, 2014).

The ability to acquire intracellular heme and hemolysin, which is based on the expression of iron uptake-associated genes [e.g., Chu heme uptake system (88.5%; 54/61), yersiniabactin (86.9%; 53/61), and aerobactin (65.6%; 40/61)], is an effective strategy for iron utilization during infection (Fischbach et al., 2006) and is another important virulence-associated feature in the repertoire of these bacteria.

### Mobile Genetic Elements

We next investigated the occurrence and circulation/transfer of ESBL-plasmids among strains and thus their contribution to the spread of antibiotic resistance in the Rwandan setting.

In total, 107 strains (89.5%; 107/120) carried plasmids with incompatibly (Inc) group FIB, followed by IncFIA (75.8%; 91/120) and IncFII (70.8%; 85/120). In particular IncF plasmids are frequently associated with genes encoding ESBL-genes, other resistances as well as virulence features important for iron acquisition (Han et al., 2012), serum resistance (Ranjan et al., 2018), and biofilm formation (Schaufler et al., 2016a).

**FIGURE 4:**
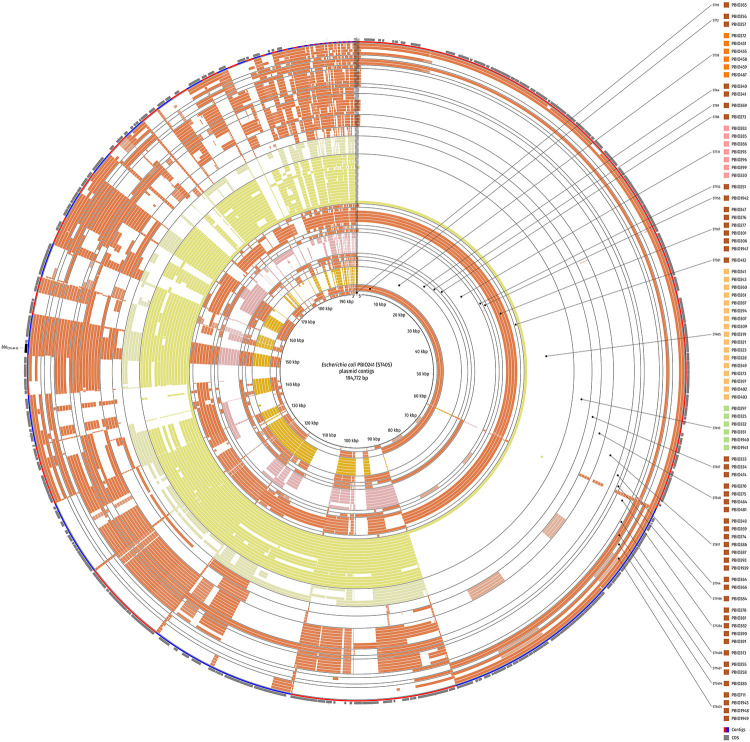
Circular comparison of the plasmid background of strains with plasmid-borne *bla*_CTX–M–15_ gene. The BLAST-classified plasmid contigs were searched against plasmid contigs of PBIO241 (ST405) as a reference. Strains are ordered by ST and name in ascending order and depicted in concentric rings from inner to outer. Red and blue arcs show contig boundaries and the outermost ring contains coding sequences (CDS; gray arrows) with the *bla*_CTX–M–15_ gene highlighted in black. The comparison was created with BLAST Ring Image Generator (BRIG).

To better assess similar plasmid backgrounds, we compared the plasmid sequences of all strains that carried a plasmid-borne *bla*_CTX–M–15_ gene against the plasmid sequence of PBIO241 (ST405; patient discharge; study-ID 159; Figure 4) as a representative for the most prevalent ST. Keep in mind, however, that the selection of the reference biases the visual representation when a large number of plasmid sequences is absent in the query sequences. On the other hand, plasmid sequences, not present in the reference but in queries, are missing in this approach. The plasmid backbones of other ST405 strains were highly similar to the reference. In contrast, plasmids from strains of other pandemic STs (ST131 and ST410) only showed few similarities when compared to the reference plasmid underlining their genetic diversity. Interestingly, in addition, the plasmid sequences of four ST167 strains [PBIO276 (patient discharge, study-ID 78), PBIO277 (patient admission, study-ID 78), PBIO301 (caregiver discharge, study-ID 131), and PBIO306 (patient discharge, study-ID 131)] were highly similar to the reference. Five other strains [PBIO256 (ST12, patient admission, study-ID 40), PBIO257 (ST12, patient discharge, study-ID 40), PBIO258 (ST1421, patient admission, study-ID 40), PBIO1948 (ST5474, housefly), and PBIO1949 (ST5474, housefly)] showed identical plasmid sequences in parts of the BRIG visualization that were absent in most of the other sequences.

We also compared some of our plasmid sequences of representative strains (criteria: top BLAST-hits) against a publicly available reference plasmid pIV_IncHI2_CTX_M_15 (Marchetti et al., 2020; Supplementary Figure 3). This *bla*_CTX–M–15_-containing plasmid has been originally obtained from an *E. coli* ST58 strain causing a deadly puppy infection in Italy in 2019. Interestingly, the plasmid backbone of PBIO251 (ST155, patient admission, study-ID 153) was nearly identical when compared to this external Italian plasmid. The plasmids of PBIO275 (ST540, caregiver admission, study-ID 78), PBIO385 (ST3494, patient discharge, study-ID 434), and PBIO1943 (ST5474, housefly) also showed high similarities to the reference and thus among each other.

The presence of similar resistance plasmids in strains of distinct sequence types and sample groups indicates the potential transmission of mobile genetic elements and the growing prevalence of successful plasmid families (Carattoli, 2011).

## Discussion

In contrast to Europe, the United States of America and Australia, only little information is available concerning the exact characteristics and distribution of ESBL-producing *E. coli* in Africa. This study reveals the broad occurrence and potential circulation of several international high-risk clonal lineages in a hospital and associated locations in Rwanda, following up on a publication by Kurz et al. (2017). Despite the potential bias due to the analysis of both admission and discharge strains of the same patient and/or caregiver, it is remarkable that our sample set was dominated by ST131, ST648, and ST410, which have been frequently reported from humans, animals and the environment (Nicolas-Chanoine et al., 2008; Ewers et al., 2010, 2014; Hussain et al., 2012; Schaufler et al., 2016b, 2019) and which are “classic” pandemic, high-risk clonal lineages. In addition to these, we found ST354 to be the third most detected sequence type in this study. Its emergence has been previously described globally except for the African continent (Manges et al., 2019). This locally restricted accumulation of one single ST in combination with a small number of other STs indicates re-entering and circulation of dominant bacterial lineages in the Rwandan hospital and among family members/neighbors and animals.

Interestingly, within the ST-associated clades, some genomes without genetic differences were interspersed in humans (hospitalized patients and caregivers as well as community members) and animals. This putative lack of host adaptation and the close phylogenetic relationships indicate the colonization and rapid transmission of several clones within the community and the potential transmission into the clinical setting and vice versa, underlined by the high acquisition rates of ESBL-producing *E. coli* during hospitalization as described previously (Kurz et al., 2017).

The resistance genes found in this study confer resistances to antibiotics frequently used in veterinary medicine and/or in sub-therapeutic doses as food supplements and growth promotors in Africa (Eagar et al., 2012; Adesokan et al., 2015; Mainda et al., 2015; Manishimwe et al., 2017). When also considering the zoonotic character of ESBL-producing *E. coli*, it is not surprising that we found clonal strains with similar patterns of resistance features in the different sample groups. Transmission likely occurred among patients and caregivers/family members and was also influenced by livestock animals due to close human-animal contact (Klous et al., 2016).

Two strains of this study carried the *mcr-9* gene but were phenotypically susceptible to colistin. This phenomenon was first reported in 2019 (Carroll et al., 2019; Kieffer et al., 2019). Due to the structural heterogeneity compared to other *mcr* genes (65% amino acid identity with the most closely related *mcr-3* gene) and the weak inactivation of colistin, the clinical importance of mcr-9 is unknown (Tyson et al., 2020).

Notably, some of the strains showed extensive, chromosomally encoded virulence-associated features. The CNF-1-encoding gene (*cnf1*) detected in strain PBIO350 (ST648), for example, is associated with causing neonatal meningitis (Khan et al., 2002). In addition to the major virulence factors of meningitis-associated and uropathogenic *E. coli* (like P fimbriae adhesion cluster, K1 capsule, heme utilization systems, and the secreted autotransporter toxin), the strains showed VAGs typical for InPEC (especially the various bacterial secretion systems) underlining the clinical relevance of these pathogens.

Finally, we demonstrate that similar plasmid sequences were present in strains from different sample groups, thus likely indicating mobile genetic element transmission, and underlining the importance of plasmid-driven spread of antimicrobial resistance independent of the host’s phylogenetic background (Schaufler et al., 2016a; Ranjan et al., 2018). Interesting in addition to similarities among strains from the Rwandan setting is in particular the close relationship to an external plasmid, which has been obtained only recently (Marchetti et al., 2020). This highlights the sometimes global spread of such mobile genetic elements and their bacterial hosts.

## Conclusion

In this study, we investigated and identified the presence of clinically relevant ESBL-producing *E. coli* that circulate among patients, caregivers, the community and animals in a Rwandan setting. The findings contribute to the understanding of the global dissemination of bacterial high-risk clonal lineages, their virulence features as well as plasmid transmissions. They also underline the potential role of houseflies in this harmful dynamic.

## Data Availability

The data for this study have been deposited in the European Nucleotide Archive (ENA) at EMBL-EBI under accession number PRJEB42795 (https://www.ebi.ac.uk/ena/browser/view/PRJEB42795).

## Author Contributions

KS and EE designed the study. EE and JM performed the laboratory and phenotypic experiments. SH, SS, and KK performed the bioinformatics analyses. KS, EE, SH, KK, CB, JN, AS, JG, MK, FM, and SS analyzed the data. KS, EE, and SH wrote the manuscript and prepared the tables and figures. All authors read and approved the final version of the manuscript.

## Conflict of Interest

The authors declare that the research was conducted in the absence of any commercial or financial relationships that could be construed as a potential conflict of interest.

## Footnotes

1 https://sourceforge.net/projects/bbmap/

2 https://github.com/tseemann/shovill/

3 https://github.com/tseemann/mlst/

4 https://github.com/tseemann/abricate/

5 https://github.com/tseemann/snippy/
