# Supplementary material for: Circulation of Extended-Spectrum Beta-Lactamase-Producing Escherichia coli of Pandemic Sequence Types 131, 648, and 410 Among Hospitalized Patients, Caregivers, and the Community in Rwanda
Source: Front Microbiol. 2021 May 14;12:662575. doi: 10.3389/fmicb.2021.662575 (PMC8160302; doi:10.3389/fmicb.2021.662575)
Supplement: Supplementary file 1 [file Data_Sheet_1.DOCX]

Supplementary Material


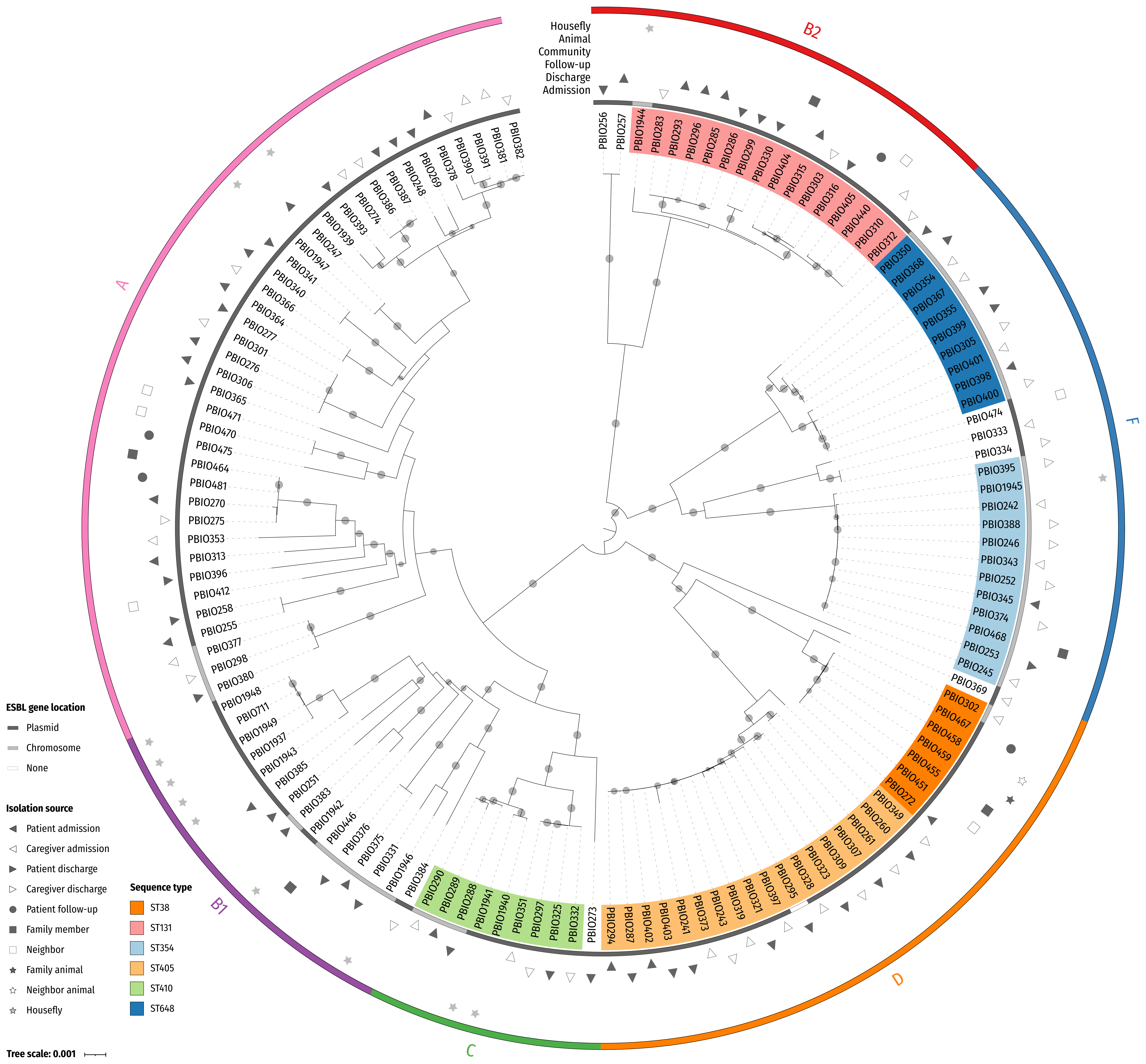


Figure S1: Midpoint-rooted whole genome-based phylogenetic tree*.* The genomes of ESBL-strains carried by houseflies (n = 13; Heiden et al., 2020b) were included. The strains belonging to the six most prevalent sequence types (STs) are highlighted in different colors. The inner circle indicates ESBL-genes encoded either on plasmids (black) or the chromosome (gray). Note that we did not detect an ESBL-gene in the PBIO295 genome *in silico* (white). In addition, different symbols and shades of gray indicate origin of strains. The outer circle shows distinct phylogroups (different colors). Shown on the branches is the rate of elementary quartets (REQ) for values ≥ 0.5 (circle diameter).


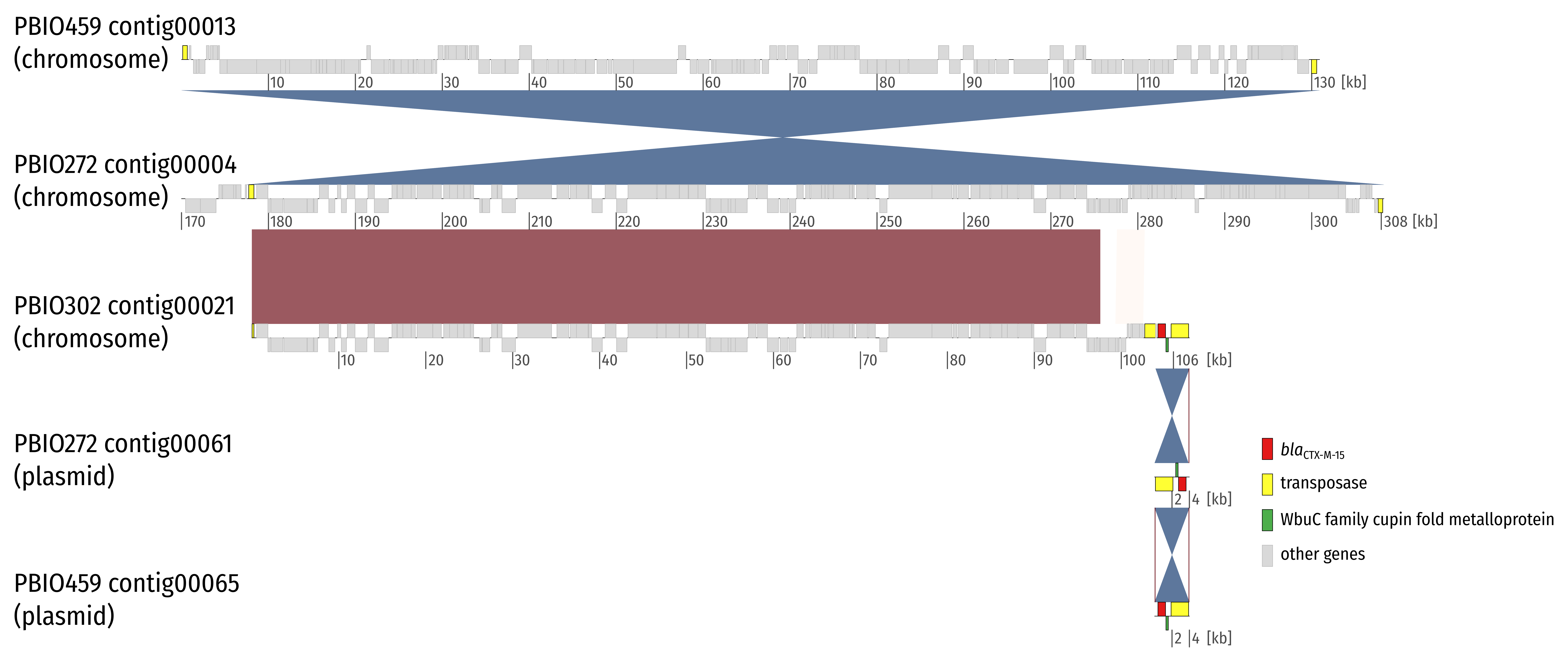


Figure S2: Synteny plot of ST38 strains PBIO272 (patient admission, study‑ID 60), PBIO302 (caregiver admission, study‑ID 131) and PBIO459 (family animal, study‑ID 133). Shown are BLASTN Megablast comparisons (*E* value 1e-10) between chromosome- and plasmid-derived contigs. Direct comparisons are colored with red hues whereas reverse comparisons are colored with blue hues. Note that PBIO302 harbors the *bla*_CTX-M-15_ gene on the chromosome whereas the other two strains (like the rest of the ST38 strains investigated in this study) carry the ESBL-gene on a plasmid.





Figure S3: Circular visualization of representative plasmid sequences (selection criteria: high similarity) compared against the sequence of plasmid pIV_IncHI2_CTX_M_15 (GenBank accession number: MN540571.1; Marchetti et al. 2020) as a reference. The GC content and GC skew are indicated on the inner ring. The matches of the sequences are presented with different colors as indicated, depending on the sequence type (ST). Gray arrows indicate the direction and size of open‑reading frames (ORFs). The *bla*_CTX-M-15_ gene is highlighted in red and (metal-) resistance genes, plasmid partitioning genes, conjugational transfer genes as well as replication initiation genes are highlighted in black. The comparison was created using BLAST Ring Image Generator (BRIG).
